# Alpha-1 Antitrypsin Deficiency Alleles in Non-Cirrhotic Hepatocellular Carcinoma: Insights from a Multicenter French Cohort

**DOI:** 10.1016/j.jceh.2026.103545

**Published:** 2026-04-03

**Authors:** Aurélie Beaufrère, Celine Leon, Marie Decreaker, Laurent Possenti, Manon Evain, Audrey Coilly, Carolin V. Schneider, Zoé Feurer, Marie-Françoise Odou, Malika Balduyck, Audrey Payancé, Kai M. Schneider, Paulette Bioulac, Jean-Frédéric Blanc, Brigitte Le Bail, Catherine Guettier, Samuel Amintas, Marion Bouchecareilh

**Affiliations:** ∗ AP-HP Nord, Department of Pathology, FHU MOSAIC, SIRIC InsiTu, DMU DREAM, Université Paris Cité, Beaujon Hospital, Clichy, France; † University of Bordeaux, CNRS, INSERM, BRIC, U1312, Bordeaux, France; ‡ Oncology Unit, Hôpital Haut Lévêque, CIC 1401, Bordeaux University Hospital, 33604, Pessac, France; § Centre Hépato-Biliaire, Hôpital Paul Brousse, UMR-1193, APHP, Université Paris Saclay, Villejuif, France; ¦ Department of Gastroenterology, Metabolic Diseases and Intensive Care, University Hospital RWTH Aachen, Aachen, Germany; ¶ CHU Lille, Department of Biochemistry and Molecular Biology ‘Hormonologie, Métabolisme-Nutrition, Oncologie’, Lille, France; # AP-HP, Hôpital Beaujon, Service d’Hépatologie, DMU DIGEST, Centre de Référence des Maladies Vasculaires du Foie, FILFOIE, ERN RARE-LIVER, Clichy, France; ∗∗ Pathology Department, Pellegrin University Hospital, CHU Bordeaux, France; †† French National and Bordeaux Local Liver Tumor Bank, France; ‡‡ Tumor Biology and Tumor Bank Laboratory, CHU Bordeaux, Pessac, France

**Keywords:** Alpha-1 antitrypsin deficiency, hepatocellular carcinoma, non-cirrhotic liver, genetic predisposition to cancer, AATD allele variants

## Abstract

**Background & aims:**

Patients with alpha-1 antitrypsin deficiency (AATD) have a 20- to 50-fold higher risk of developing primary liver cancer (PLC), such as hepatocellular carcinoma (HCC), in both cirrhotic and noncirrhotic livers, highlighting AATD as a potential oncogenic factor. In this exploratory study, we aimed to describe the frequency and characteristics of AATD alleles in patients with HCC arising in noncirrhotic livers.

**Methods:**

The two most common pathogenic AATD variants, the Z (SERPINA1 p.Glu366Lys) and S (SERPINA1 p.Glu264Val) alleles, were identified using a multiplex digital polymerase chain reaction (PCR) system in a French cohort of 91 patients who developed HCC in a noncirrhotic liver and without major liver risk factors, including alcohol consumption, viral hepatitis, or metabolic-associated steatohepatitis.

**Results:**

We found that 22.83% (21/91) of patients with HCC carried at least one S or Z allele of *SERPINA1*, a prevalence that was higher than in the general French population (16.9%) and in the UK population with PLC (12%). The distribution of genotypes was heterozygous for the M and S alleles (18/21), heterozygous for the M and Z alleles (2/21), and homozygous for the S allele (1/21). Interestingly, AAT globules were observed in nontumoral liver tissue in 7 cases (37%), including individuals heterozygous for the M and S alleles and the individual homozygous for the S allele.

**Conclusion:**

Our study provides descriptive data on the presence of S and Z variants of *SERPINA1* in patients with HCC arising in noncirrhotic livers, highlighting their potential relevance for further investigation.

**Clinical trial number:**

NCT07145385.

## Graphical abstract

**Figure:**
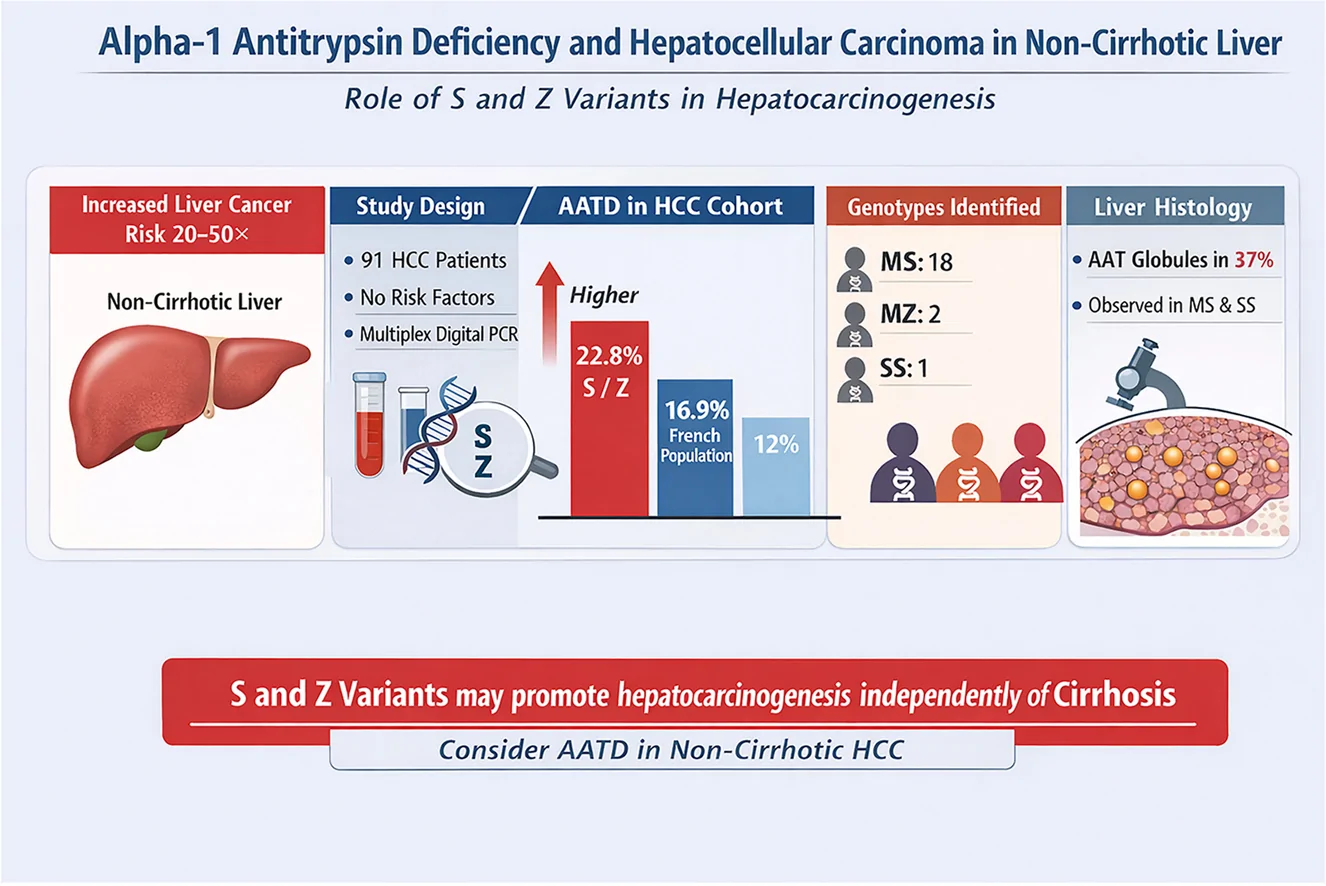

Hepatocellular carcinoma (HCC) is the most common primary liver cancer (PLC) and a leading cause of cancer-related mortality worldwide.^1^ It typically develops in a cirrhotic liver (80% of cases), while less than 20% of HCC cases arise in a noncirrhotic liver.^2^ However, cirrhosis remains the strongest risk factor for HCC.^1^ Other risk factors include viral hepatitis, alcohol-related liver disease, diabetes, obesity-related metabolic dysfunction-associated steatohepatitis (MASH), and genetic conditions such as alpha 1-antitrypsin deficiency (AATD).^3^^,^^4^

AATD is a rare inherited disorder characterized by reduced levels of the AAT protein in the blood, which predisposes affected individuals to complications, particularly those involving the lungs and liver.^5^ This condition is caused by *SERPINA1* mutations, most commonly the S (rs17580) and Z (rs28929474) allele variants.^6^^,^^7^ S and Z alleles are present in all European countries, though their distribution varies significantly, even within different regions of the same country^8^, ^9^, ^10^. The Z allele is most frequent in north-western Europe. Its frequency ranges from 7/1000 in Switzerland to 27.9/1000 in Denmark^8^, ^9^, ^10^. In contrast, the S allele peaks in the Iberian Peninsula (100–200/1000) and drops to 19/1000 in Northern Ireland.^8^, ^9^, ^10^.

The Z allele variant is the most clinically severe, accounting for 95% of cases with clinical symptoms,^6^^,^^7^ and is associated with both pulmonary and hepatic manifestations. The pathogenicity of the Z allele variant arises from a single point mutation that induces protein misfolding, leading to its retention and aggregation within the endoplasmic reticulum (ER) of hepatocytes.^6^^,^^7^ When these aggregates accumulate excessively, they become sequestered within intracellular globules, which can be histologically visualized by a periodic acid-Schiff (PAS)-positive, diastase-resistant staining.^11^ These structures can trigger proteotoxicity and activate stress pathway cascades, contributing to progressive liver diseases (chronic liver disease, cirrhosis, and HCC).^6^^,^^7^^,^^12^

In contrast to chronic liver disease and cirrhosis, research focusing on AATD-related liver cancers remains limited. Results obtained from Northern European cohorts showed that the prevalence of HCC in individuals homozygous for the Z allele is about 2%, with a 23.4-fold higher risk of developing hepatic cancer (95% confidence interval [CI], 9.88–55.39) compared to the general population.^13^^,^^14^ A distinctive feature of ZZ patients is that they can develop HCC on both cirrhotic and noncirrhotic livers,^14^^,^^15^ suggesting that the Z allele itself may contribute to hepatic carcinogenesis. However, no studies to date have demonstrated this by specifically assessing the isolated effect of the Z allele while excluding other known PLC risk factors.

In addition, all existing studies were conducted in regions where the S allele is rare, even though growing evidence suggests that homozygosity for the S allele may also be pathogenic.^16^^,^^17^ Several studies have linked individuals homozygous for the S allele (*SERPINA1* p.Glu264Val) to chronic obstructive pulmonary disease (COPD),^18^^,^^19^ and findings from the EARCO project by Martin *et al.* showed that although individuals homozygous for the S allele have a lower risk of lung damage compared with individuals homozygous for the Z allele, their risk is comparable to that of SZ heterozygotes despite higher circulating AAT levels.^17^ Similar results were reported by *Fraughen et al.*^16^ In a 2023 ATS conference abstract, the authors noted that in individuals homozygous for the S allele individuals with a smoking history were more prone to emphysema. They also reported that 68% had abnormal liver function tests (LFTs) at diagnosis, 21% had LFTs >3 × ULN, and that the median liver stiffness among non-liver-related referrals was 11.23 kPa, consistent with F3 fibrosis.

Taken together, these findings suggest that the S allele variant may be more detrimental than previously assumed and could play a role in AATD-related liver injury, including HCC. France, characterized by a high genotypic diversity of AATD variants, therefore provides a unique setting in which to explore the potential contribution of these alleles to HCC development.

To explore the occurrence of AATD variants in HCC, we conducted a retrospective French study analyzing a cohort of patients with HCC arising in noncirrhotic livers and without major liver risk factors, including alcohol consumption, viral hepatitis B or C, and MASH, stratified by AATD genotype.

## MATERIALS AND METHODS

### Study Population Selection and Collected Data

We conducted a retrospective study of all HCC cases diagnosed at Bordeaux University Hospital and Paris Beaujon Hospital (France) for which Formalin-fixed paraffin-embedded tissue (FFPE) samples were available. Among the 3074 patients diagnosed between January 2005 and December 2021 in Bordeaux, and between January 2012 and December 2022 in Beaujon, 279 had HCC with no known predisposition (i.e. no history of alcohol consumption, viral hepatitis, metabolic syndrome, hemochromatosis, or autoimmune disease) (Figure S1). Among these patients, 188 were excluded because they developed HCC on a cirrhotic liver or due to a lack of informed consent for genetic testing or insufficient tumor tissue (Figure S1). Ultimately, 91 patients with HCC on a noncirrhotic liver and without a known predisposition were included. Collected data included demographic variables (gender, age, body mass index (BMI)), tumor characteristics (tumor size, number of initial hepatic tumors, metastasis status, recurrence status, Edmondson grade, fibrosis stage), clinical information (mode of discovery, surgical procedure date, recurrence date), and survival outcomes (date of death and date of the last follow-up). The study was approved by the local ethics committees (CER-BDX 2023-101) and conducted in accordance with the rules of good practice in the European Union and the ethical principles of the Declaration of Helsinki. The use of biologic data was authorized by the Biological Resource Centre, Centre de Ressources Biologiques (CRB) Paris Sud and Bordeaux.

### Population-Based Data

Data on the prevalence of *SERPINA1* S and Z alleles in the French population and in a U.S. cholangiocarcinoma cohort were extracted from published sources (*Blanco et al.*^9^ and *Cornillet et al.*,^20^ respectively). Regarding the “UK Biobank” (UKB), it is a large population-based prospective cohort conducted in the United Kingdom that recruited 502,511 participants aged 37–73 years at baseline. All participants provided written informed consent and underwent baseline assessments, including genotyping and linkage with routinely collected health records. Inpatient hospital data available since 1996 were used to identify diagnoses coded according to the International Classification of Diseases, 10th revision (ICD-10). Genotype data for the *SERPINA1* Z (rs28929474) and S (rs17580) alleles were available for 487,503 participants. Individuals with viral hepatitis (ICD-10 B16–B19; 713 MM, 20 MZ, 2 SZ) or excessive alcohol consumption (>60 g/day for men and >40 g/day for women; 3775 MM, 153 MZ, 9 SZ, 3 ZZ, 4 SS) were excluded. Primary liver cancer was defined using ICD-10 code C22.0. The UKB study was approved by the North West Multicenter Research Ethics Committee (REC reference: 16/NW/0274) and this study was approved under the UKB access number 71300.

### Exclusion of Risk Factors and Fibrosis Assessment

All patients were systematically evaluated to exclude major liver disease risk factors, including MASH, alcohol consumption, hepatitis B and C virus infection, hemochromatosis, and autoimmune liver disease. MASH was assessed using comprehensive clinical, biological, and histological data. Hepatitis B and C virus status was available for all patients, and individuals with chronic viral hepatitis were excluded. Alcohol consumption was assessed based on patient self-report, using exclusion thresholds of >60 g/day for men and >40 g/day for women. Hemochromatosis and autoimmune liver diseases were excluded based on available clinical history, biological markers, and histological features. Liver histology was available for all patients, and fibrosis in nontumoral liver tissue was evaluated according to the METAVIR staging system to confirm noncirrhotic status. All assessments were performed in specialized hepatology–gastroenterology units, with histological evaluation conducted by specialized liver pathologists. Relevant data were obtained from routine clinical care, including imaging and laboratory reports, ensuring a well-characterized cohort with minimized confounding from major metabolic, viral, genetic, or immune-mediated liver diseases.

### Samples and DNA Extraction

Patients' FFPE liver tissue samples were obtained from the Cancer Biological Resource Center at Bordeaux University Hospital and Liver Cancer Biological Resource Center. FFPE sample blocks included both tumor and nontumor tissue, except for four cases where only tumor tissue was available. DNA was extracted from tissue sections obtained from the paraffin block using the Maxwell RSC DNA FFPE Kit (Promega, France) at the Tumor Biology Department of Bordeaux University Hospital. Extracted DNA was eluted in 70 μl of “nuclease-free” water and DNA concentration was determined by fluorimetry with DS11FX automated system (DeNovix, United States).

### Digital PCR Design and Analysis

The allele statuses of the Z and/or S allele variants in the *SERPINA1* gene were determined using a custom-designed multiplex digital polymerase chain reaction (PCR) assay, performed on the Qiagen QIAcuity One® system. The reactions were carried out on 24-well plates, with each well partitioned into approximately 8500 individual reactions. A total of 50 ng of genomic DNA was analyzed per patient. The list of primers, probes, and fluorophores used for the assay is provided in Table S1. A positive internal control was generated using a plasmid incorporating the S (exon 4) and Z (exon 5) allele variant sequences of *SERPINA1*, along with a puromycin resistance gene sequence. The plasmid was subsequently integrated into lentiviral particles (vector map available in Figure S2A). Immortalized human fibroblasts were transduced with the lentivirus and selected with puromycin (5 μg/mL for 72 h). A cell pellet was then collected, fixed in 10% formalin, and embedded in paraffin. Tissue sections from the paraffin block were used for DNA extraction. The same process was applied to generate a negative control using wild-type immortalized human fibroblasts. Both negative (wild-type DNA) and positive (synthetic DNA carrying the S and Z variants) internal controls were included in each dPCR run (Figure S2B).

### Histology

A PAS-diastase staining was performed on the available AATD samples (n = 19) using a tissue section taken from the interface between tumor and nontumoral tissue. All histological slides, including hematoxylin–eosin–saffron (HES) and PAS-diastase, were reviewed by a liver pathologist (A.B.). The following criteria were assessed: tumor grade (according to the World Health Organization [WHO] classification^21^); histological subtype (according to the WHO classification, including steatohepatitic, clear cell, macrotrabecular-massive, scirrhous, chromophobe, neutrophil-rich, and lymphocyte-rich subtypes, or HCC not otherwise specified (NOS)); presence of the vessels-encapsulating tumor clusters (VETC) phenotype; vascular invasion; tumor fibrosis; inflammatory infiltrate; intracellular globules on PAS-diastase staining (assessed in both tumor and nontumoral areas); and fibrosis in the nontumoral liver, evaluated according to the Metavir staging system.^22^

### Statistical Analysis

All continuous variables were analyzed using unpaired Mann–Whitney U tests and are presented as mean ± standard deviation (SD). Categorical variables were compared using Fisher's exact test. Survival analyses were conducted using Cox regression analysis. Overall survival (OS) was defined as the time from the date of curative-intent surgical resection to the date of death from any cause or to the date of the last follow-up for patients still alive. Recurrence-free survival (RFS) was defined as the time from the date of curative-intent surgical resection to the date of first documented tumor recurrence or to the date of the last follow-up for patients without recurrence. A *P*-value <0.05 was considered statistically significant. All statistical analyses were performed with RStudio (R Foundation for Statistical Computing, Vienna, Austria) and GraphPad Prism version 8 (GraphPad Software, La Jolla, CA, USA).

## RESULTS

### Study Population Description

The cohort included 52 males (57.1%) and 39 females (42.9%), with a mean age of 62.9 years (SD 14.3) and a mean body mass index (BMI) of 25.0 kg/m^2^ (SD 4.3). Hepatic tissue fibrosis staging revealed F0 in 51 patients (56.7%), F1 in 29 (32.2%), F2 in 6 (6.7%), and F3 in 4 (4.4%). The majority of cases were discovered incidentally (37/91), while others presented with pain and discomfort (28/91) or abnormal biological analyses (16/91). Tumor characteristics showed an average size of 90.4 mm (SD 48.1). According to the WHO grade, 43 patients (47.8%) had grade 1 tumors, 8 (8.9%) had grade 2, and 39 (43.3%) had grade 3. Metastases were absent in 74 patients (82.2%), while 16 (17.8%) showed metastatic spread. A single hepatic tumor was observed in 79 patients (86.8%), and multiple tumors in 12 (13.2%). Recurrence occurred in 52 patients (57.1%), whereas 39 (42.9%) had no recurrence. All clinicopathological data are summarized in Table 1.

**Table 1:** Descriptive Statistics of Cohort Clinicopathological Features.

Variable	All Patients (n = 91)		
	Mean ± SD		
Age (years)	62.9 ± 14.3		
BMI (kg/m^2^)	25.0 ± 4.3		
Tumor size (mm)	90.4 ± 48.1		

**Variable**	**Category**	**Count**	**Percentage**
Sex	F	39	42.9
	M	52	57.1
Fibrosis stage	0	51	56.7
	1	29	32.2
	2	6	6.7
	3	4	4.4
Discovery	LA	16	18.0
	GHI	8	9.0
	DP	28	31.5
	FD	37	41.6
Number of tumors	1	79	86.8
	2+	12	13.2
Metastasis	Yes	16	17.8
	No	74	82.2
Recurrence	Yes	52	57.1
	No	39	42.9
WHO grade	1	43	47.8
	2	8	8.9
	3	39	43.3
AATD genotype	MM	70	76.9
	MS	18	19.8
	MZ	2	2.2
	SS	1	1.1

AATD, alpha-1 antitrypsin deficiency; F, female; M, male; LA, laboratory abnormality; GHI**,** general health impairment; DP, discomfort and pain; FD, fortuitous discovery. BMI, body mass index; SD, standard deviation; WHO, World Health Organization.

### AATD dPCR Genotyping

**Figure 1:**
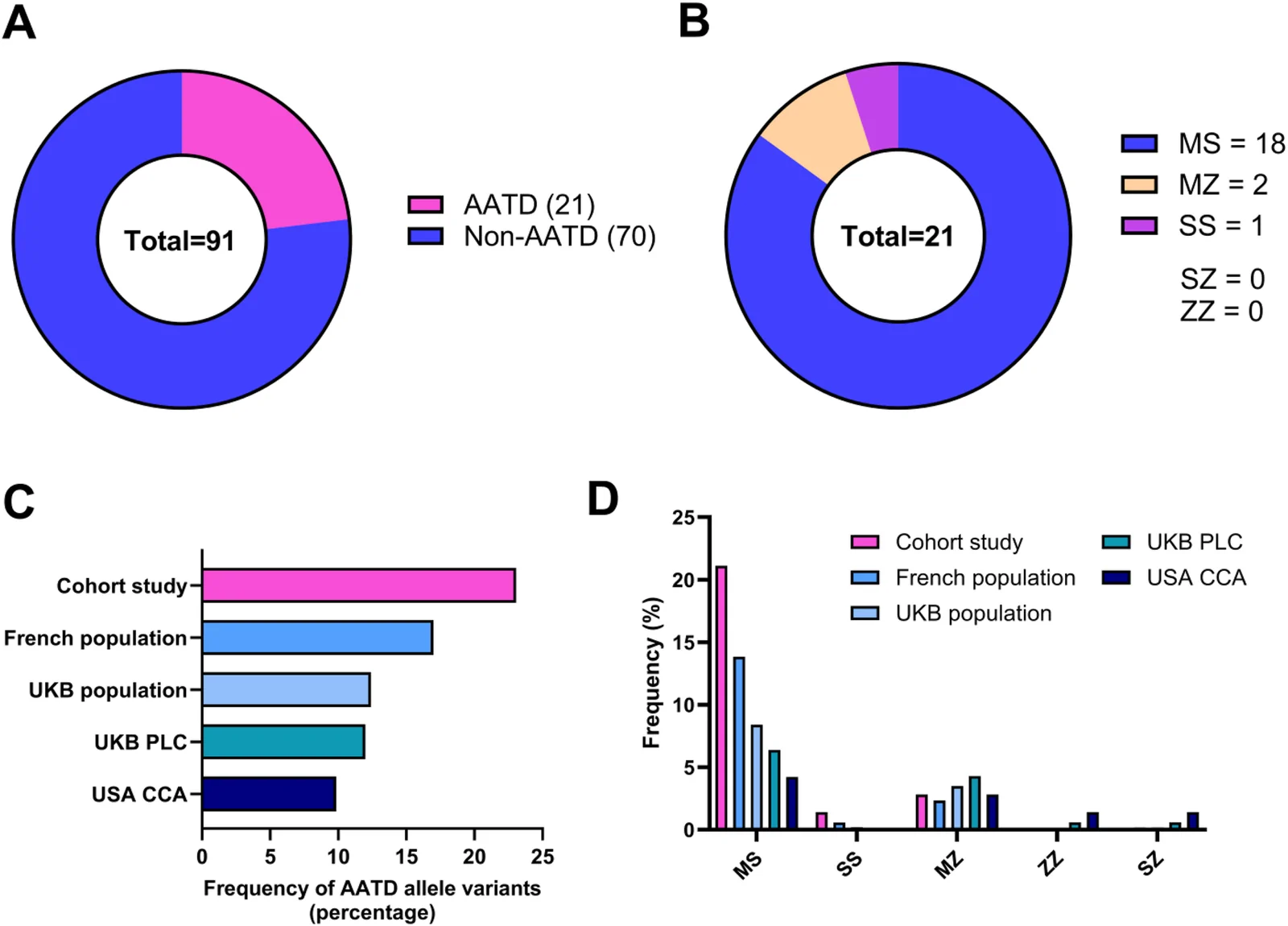
**AATD allele variants in patients with HCC in non-cirrhotic liver.** (**A**) Distribution of AATD allele variants within the study cohort. (**B**) Genotype distribution of AATD subjects in the study cohort. (**C**) Frequency of AATD allele variants observed in the study cohort compared with frequencies reported in published population-based screening studies. (**D**) Distribution of AATD genotypes in the study cohort compared with distributions reported in published population-based screening studies. AATD, alpha-1 antitrypsin deficiency; HCC, hepatocellular carcinoma.

AATD genotyping of the 91 cases identified 21 patients carrying at least one Z or S allele (MS = 18, MZ = 2, SS = 1), hereafter referred to as AATD subjects (Figure 1A–B). Compared to retrospective datasets (UKB,^9^^,^^20^) the prevalence of AATD subjects’ in our cohort was higher than that observed in the general French population (16.9%), the general UK population (12.41 %), as well as in CCA patients (9.86%) and in individuals with primary malignant neoplasms of the liver (PMNL) (12%, Figure 1C), with the MS genotype prevalence significantly higher for all studies except the general French population (Figure 1D).

### Association of AATD Status with Clinicopathological and Survival Features

**Figure 2:**
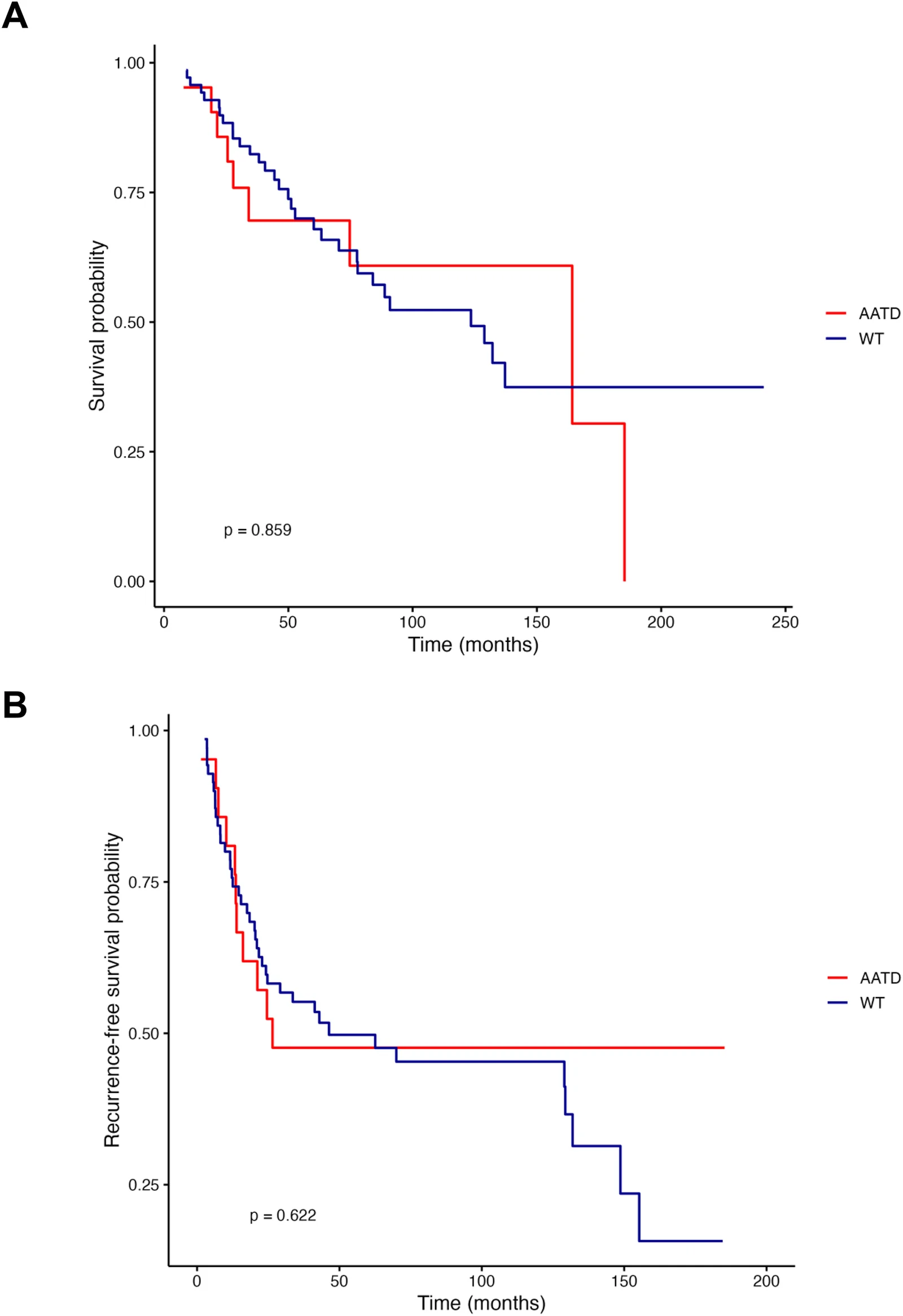
**Comparative analysis of overall and recurrence-free survival in patient cohorts with and without AATD allele variant.** Cox regression analysis of (**A**) overall survival and (**B**) recurrence-free survival, according to the presence of AATD allele variants. AATD, alpha-1 antitrypsin deficiency.

We next examined whether HCC associated with AATD exhibited specific clinical, pathological, or survival features (Table 2). The only significant difference was observed for gender distribution (Table 2), with a higher proportion of men among AATD subjects (*P* = 0.0492). No significant differences were observed between patients with or without AATD regarding overall (*P* = 0.859; Figure 2A) and recurrence (*P* = 0.622; Figure 2B) -free survival.

**Table 2:** Comparative Clinicopathological Features of HCC Patients Regarding AATD Status.

Variable	Category	All patients (n = 91)		*P*-value
		No AATD (n = 70)	AATD (n = 21)	
Sex	Female	34 (48.2%)	5 (23.8%)	**0.0492**
	Male	36 (51.8%)	16 (76.2%)	
Age (years)	NA	62.1 ± 14.3	65.9 ± 14.4	0.454
BMI (kg/m^2^)	NA	25.1 ± 4.7	24.6 ± 2.9	0.992
Fibrosis stage	F0	36 (52.2%)	15 (71.4%)	0.3436
	F1	24 (34.8%)	5 (23.8%)	
	F2	6 (8.7%)	0 (0%)	
	F3	3 (4.3%)	1 (4.8%)	
Discovery	LA	14 (20.6%)	2 (9.5%)	0.5769
	GHI	5 (7.4%)	3 (14.3%)	
	DP	21 (30.9%)	7 (33.3%)	
	FD	28 (41.1%)	9 (42.9%)	
Tumor size (mm)	NA	92.6 ± 52	82.7 ± 30.8	0.631
Number of hepatic tumors	1	61 (87.1%)	18 85.7%)	1.0000
	2+	9 (12.9%)	3 (12.3%)	
Metastasis	No	58 (84%)	16 (76.2%)	0.5148
	Yes	11 (16%)	5 (23.8%)	
Recurrence	No	29 (41.4%)	10 (47.6%)	0.6252
	Yes	41 (58.6%)	11 (52.4%)	
WHO tumor grade	1	34 (49.3%)	9 (42.8%)	0.7009
	2	7 (10.1%)	1 (4.8%)	
	3	28 (40.6%)	11 (52.4%)	

AATD, alpha-1 antitrypsin deficiency; F, female; M, male; BMI, body mass index; LA, laboratory abnormality; GHI**,** general health impairment; DP, discomfort and pain; FD, fortuitous discovery; WHO, World Health Organization.

### Histological Features of AATD Samples

**Figure 3:**
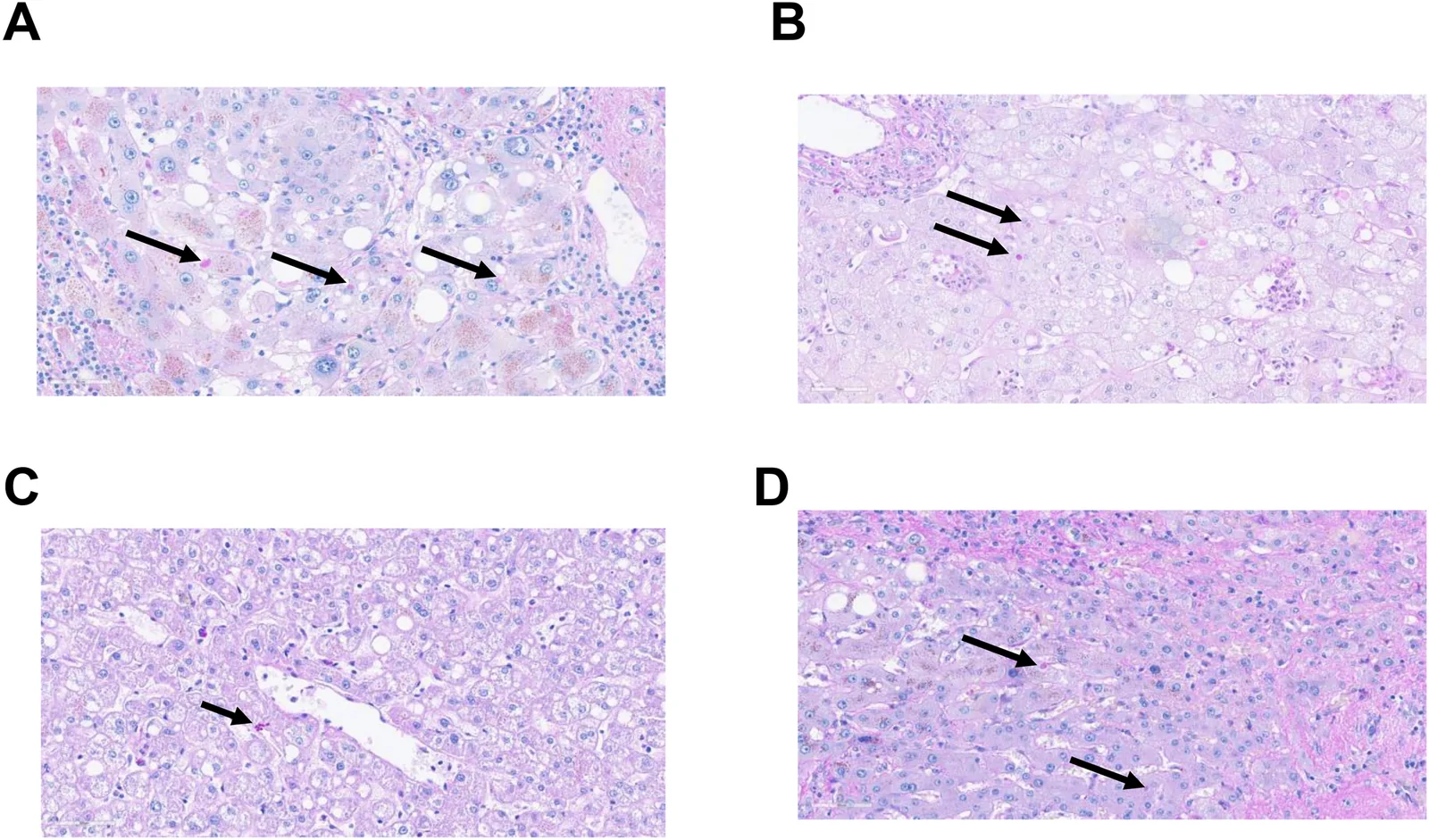
**Morphologic characteristics in PAS-diastase staining of nontumoral liver tissues in MS and SS patients.** (**A–B**) Small S allele–related globules (arrows) observed in some hepatocytes from individuals heterozygous for the MS alleles, slightly marked by PAS-diastase staining. (**C-D**) Small S allele–related globules (arrows) identified in an individual homozygous for the S allele, slightly marked by PAS-diastase staining and arranged in a chain (**C**). PAS, periodic acid-Schiff.

Histological analysis of the AATD samples revealed that the tumors were predominantly well-differentiated (42%) and classified as not otherwise specified (NOS; 63%). The VETC phenotype and vascular invasion were observed in 26% and 42% of cases, respectively. In the non-tumoral liver, most cases were classified as F0 or F1 (58%) according to the Metavir staging system (Table S2). Interestingly, intracytoplasmic PAS-diastase–positive globules were observed in the nontumoral areas in 7 cases (37%). These globules were generally very focal, weakly positive, and small in size, and none were identified within the tumor areas. This finding was observed in individuals heterozygous for the MS alleles (6 cases) and in the individual homozygous for the S allele (SS), but not in individuals heterozygous for the MZ alleles (Figure 3A–B). To exclude the possibility of other rare AAT allele variants, complete *SERPINA1* sequencing was performed on these cases, revealing no additional mutations.

### SS Homozygotes Cases

As previously mentioned, emerging evidence indicates that individuals homozygous for the S allele may be more pathogenic than initially thought, affecting both pulmonary and hepatic systems.^16^^,^^17^ Supporting this idea, we identified in our cohort a 54-year-old male homozygous for the S allele with an incidental hepatic mass, without chronic liver disease or MASH, alcohol consumption, hepatitis B and C virus infection. His history included HIV (diagnosed in 1992, treated since 1996), hypertension, allergic asthma, and hematuria. Histology showed a well-differentiated steatohepatitic HCC without vascular invasion or satellite nodules. PAS-diastase staining revealed small intracytoplasmic globules in hepatocytes arranged in chains, consistent with atypical AAT inclusions (Figure 3C).

Interestingly, we identified one patient homozygous for the S allele in our initial cohort, and, upon examining a second HCC liver transplant cohort that was entirely independent from the first in terms of recruitment and clinical context,^23^ we found another patient homozygous for the S allele. This 89-year-old male had a tumor arising on chronic precirrhotic liver disease due to metabolic dysfunction and iron overload (heterozygous *HFE* H63D). The tumor was classified as NOS HCC with clear-cell (60%) and steatohepatitic (20%) components, moderately to poorly differentiated, without vascular invasion but with a satellite micronodule. PAS-diastase staining revealed a few positive intracytoplasmic globules (Figure 3D). His AAT concentration was 1.3 g/L in 2022.

## DISCUSSION

Here, we estimate the prevalence of the Z and S alleles to be around 23% in HCC occurring in noncirrhotic livers without known predisposition. This proportion appears higher than in control populations (in particular for the French one) and in patients with PLC (HCC and CCA).

However, the majority of AATD genotypes identified in our cohort were MS, MZ, and SS. These data emphasize the geographic distribution of AATD genotypes across Europe. Indeed, the frequency of the Z allele is higher in Northern Europe, whereas the S allele is more prevalent in Southern Europe (Spain, Portugal, etc.). France is consequently well-situated to study this heterogeneity in AATD genotypes.

Interestingly, we did not identify any individuals homozygous for the Z allele or heterozygous for the S and Z alleles in our cohort. This absence may reflect the origin of our cohort (France) and the relatively small sample size (91 patients). It also highlights that any potential contribution of Z alleles to hepatocarcinogenesis cannot be assessed in this cohort and may depend on additional risk factors, such as cirrhosis, alcohol consumption, or viral hepatitis. To further explore these questions, future studies should analyze larger, unselected HCC cohorts, systematically screen for AATD alleles, and correlate the findings with detailed clinical and pathological characteristics.

Additionally, across two independent cohorts, we identified two cases of HCC in individuals homozygous for the S allele, one occurring in a cirrhotic liver and the other in a noncirrhotic liver. Unlike the Z allele, the contribution of the S allele to liver disease remains poorly defined, despite its relatively high prevalence in several European countries. As previously noted, emerging evidence suggests that individuals homozygous for the S allele may not be entirely benign. In particular, *Fraughen et al.* reported abnormal liver function tests at diagnosis in 68% of individuals homozygous for the S allele,^16^ and prior studies have also linked individuals homozygous for the S allele to COPD, with *Martin et al.* showing a risk of lung damage comparable to individuals heterozygous for the SZ alleles despite higher AAT levels^17^, ^18^, ^19^.

In pathological practice, the presence of PAS-positive, diastase-resistant globules is most commonly associated with the Z allele variant. *Matamala et al.*^24^ reported the presence of retained AAT aggregates in HEK293T cells expressing the S variant, as demonstrated by hematoxylin–eosin and periodic acid–Schiff staining. Our findings extend these observations by showing that, like the Z variant, the S variant can also form aggregates in human liver tissue. This raises important questions about the mechanism underlying polymer and aggregate formation in the S variant. Since the S variant is located in a different region of the protein than the Z variant, existing pathogenic models may not be directly applicable. Are the aggregates structurally similar to those formed by the Z variant, or do they result from additional somatic variants^25^? Are they composed exclusively of SS aggregates, or do the S allele variant form heteropolymers with normal AAT? Furthermore, is the composition of these aggregates similar to, or distinct from, the granules observed in ZZ patients? Although we did not perform 2C1 immunostaining in this study due to routine practice constraints, this monoclonal antibody, which specifically recognizes polymerized AAT while not binding to the native monomeric form, could be particularly useful in future investigations involving larger, unselected HCC cohorts to determine whether the observed inclusions correspond to classical AAT polymers.

Notably, in our histological analyses, the aggregates associated with the S variant were generally only detectable by PAS-diastase staining, appeared very focal, and stained weakly, in contrast to ZZ aggregates, which are usually numerous, visible on HES staining, and strongly PAS-diastase positive. These differences suggest a potentially distinct structural or biochemical nature of the aggregates formed by the S allele variant, raising new questions about its liver pathogenic potential and clinical impact.

In addition to these mechanistic considerations, we characterized the biological, pathological, and survival profiles of HCC tumors according to AATD allele status. The only significant difference observed in our cohort was gender distribution, with a higher proportion of men among patients with HCC and AATD. This result is in line with findings from mouse models^26^ and studies of liver fibrosis/cirrhosis,^27^ reporting an increased risk of liver damage related to AATD in males. Despite limited data, this may be linked to hormonal influences, as AAT expression is higher in male PiZ mice, and testosterone treatment of female PiZ mice increases Z-AAT protein expression to levels comparable with those in males.^28^

We acknowledge several limitations to this study, including the relatively small cohort size, the absence of a validation cohort, and the lack of a matched control population. Another important limitation is the absence of information on patient ethnicity, which may significantly influence the observed prevalence of AATD allele variants. Consequently, our findings should be validated in larger, multicenter cohorts and, ideally, integrated with current state-of-the-art knowledge on the natural history of AATD to provide deeper insights into the underlying pathobiological associations.

In conclusion, our study provides descriptive insights into the presence of S allele variants in patients with HCC arising in noncirrhotic livers. While no causal or risk associations can be established, the observed patterns suggest that carriers of AATD alleles may exhibit distinct clinicopathological features worthy of further investigation. Larger, unselected cohorts and mechanistic studies are needed to clarify the significance of these variants and to better understand their potential role in hepatocarcinogenesis.

## Ethics statement

The study was approved by the local ethics committees (CER-BDX 2023-101) and conducted in accordance with Good Clinical Practice guidelines in the European Union and the ethical principles of the Declaration of Helsinki. The use of biological data was authorized by the Biological Resource Centres (CRBs) of Paris Sud and Bordeaux. The clinical trial is registered under ClinicalTrials.gov identifier NCT07145385.

## Credit authorship contribution statement

M.B. and S.A. conceived and designed the study. A.B., C.L., M.D., L.P., M.E., A.C., A.P., C.V.S., Z.F., M.F.O, M.B., K.M.S., P.B., J.F.B., C.G., B.L.B., M.B. and S.A. contributed to the acquisition of data. A.B., C.L., M.D., M.E., A.C., C.V.S., M.F.O, M.B., B.L.B., M.B. and S.A. analyzed the data. A.B., C.L., M.D., M.E., A.C., C.V.S., M.F.O, M.B., B.L.B., M.B. and S.A. interpreted the data. A.B., M.B. and S.A. drafted, reviewed and edited the manuscript. Further authors substantially contributing to the final version of the manuscript: C.L., M.D., M.E., A.C. C.V.S. All authors read and approved the final version of the manuscript.

## Funding

This research has been conducted using the UK Biobank Resource under Application Number 71300. UK biobank data was accessed by C.V.S Copyright © 2023, NHS England. Re-used with the permission of the NHS England and UK Biobank. All rights reserved. This work uses data provided by patients and collected by the NHS as part of their care and support. This work was funded by ADAAT Alpha1-France, Orphalan, LFB Biomédicaments, CSL Behring, the University of Bordeaux (France), CNRS, and INSERM.

## Declaration of competing interest

Marion Bouchecareilh received consulting fees from Grifols Inc.
