# Supplementary material for: Alpha-1 Antitrypsin Deficiency Alleles in Non-Cirrhotic Hepatocellular Carcinoma: Insights from a Multicenter French Cohort
Source: J Clin Exp Hepatol. 2026 Apr 3;16(4):103545. doi: 10.1016/j.jceh.2026.103545 (PMC13127472; doi:10.1016/j.jceh.2026.103545)
Supplement: Supplementary file 1 [file mmc1.pdf]

Supplementary data:

Alpha-1 Antitrypsin Deficiency Alleles in Non-Cirrhotic Hepatocellular Carcinoma: Insights from a Multicenter French Cohort

Aurélie Beaufrère[1#], Celine Leon[2#], Marie Decreaker[2,3], Laurent Possenti[3], Manon Evain[4], Audrey Coilly[4], Carolin Victoria Schneider[5], Zoé Feurer[2], Marie-Françoise Odou[6], Malika Balduyck[6], Audrey Payancé[7], Kai Markus Schneider[5], Paulette Bioulac[2], Jean-Frédéric Blanc[3], Brigitte Le Bail[2,8-9], Catherine Guettier[4], Samuel Amintas[2,10&], Marion Bouchecareilh[2&].

Supplemental Figures Legends

Figure S1. Flowchart for patient selection

Figure S2. dPCR assays details. Schematic map of the pLKO1 plasmid used for lentivirus production and subsequent transduction of HEK cells, enabling the generation of a stable cell line, incorporating targeted sequences of the Z and S variants of the *SERPINA1* gene (**A**). dPCR analysis dot plots showing negative (top) and positive (bottom) controls, with the Z variant detected on the left (FAM fluorescence on the x-axis, HEX fluorescence on the y-axis) and S variant on the right (TAMRA fluorescence on the x-axis, ROX fluorescence on the y-axis) (**B**).

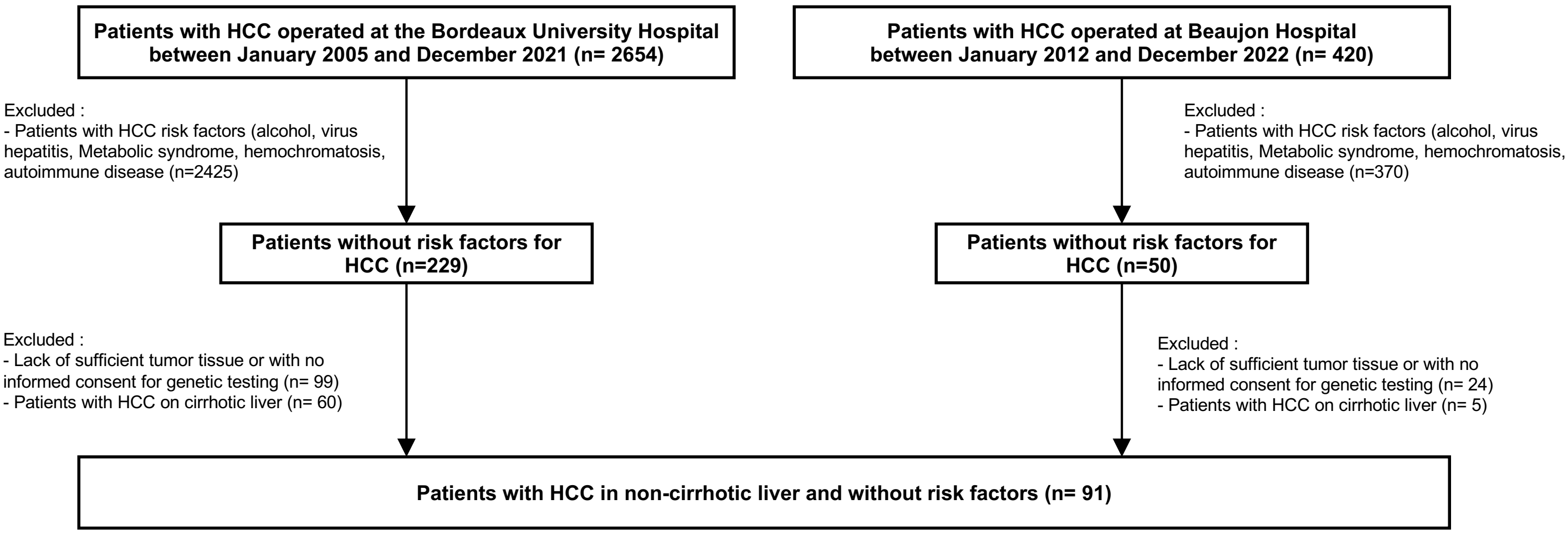

Supplemental Figure 1

A

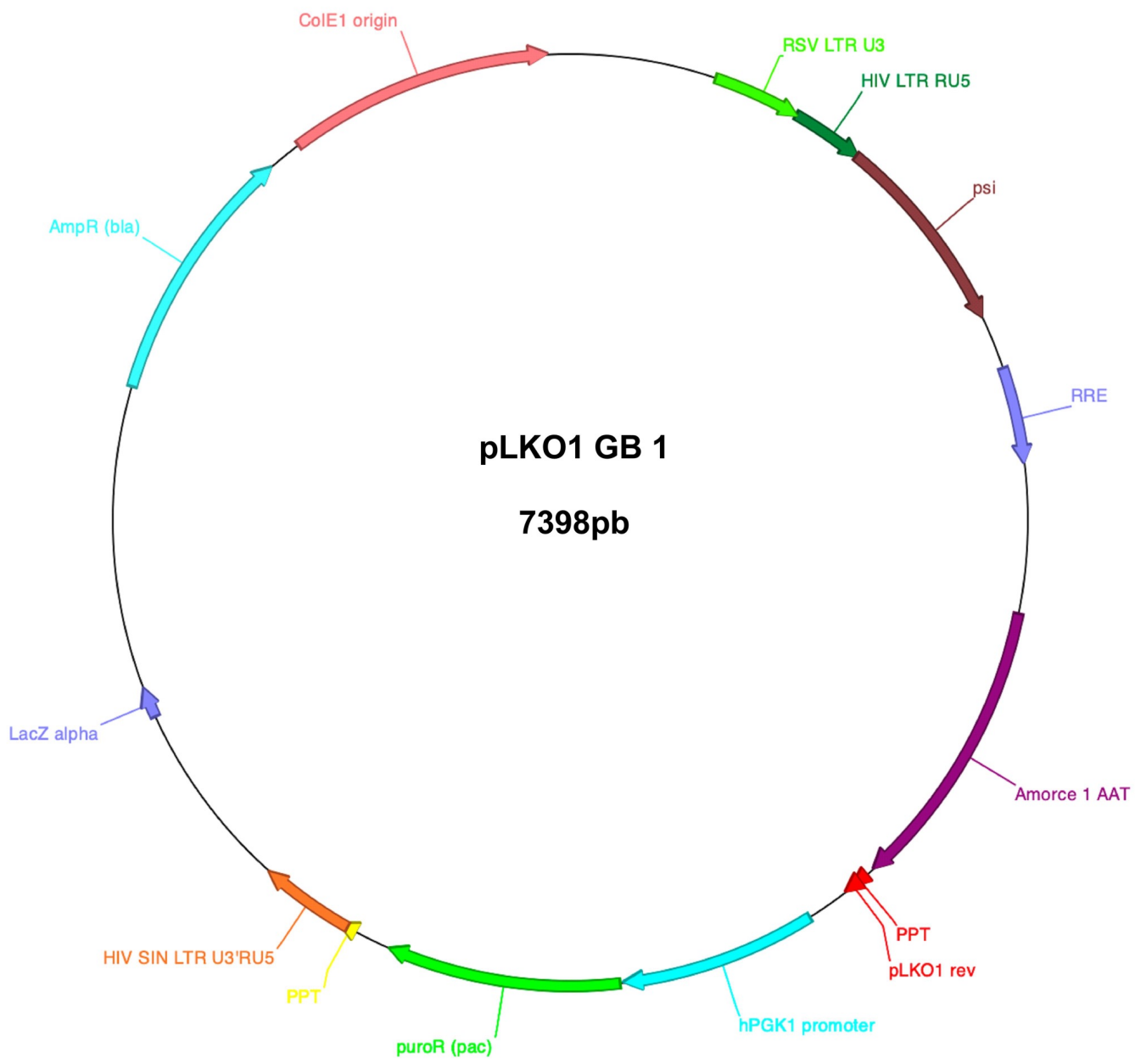

B

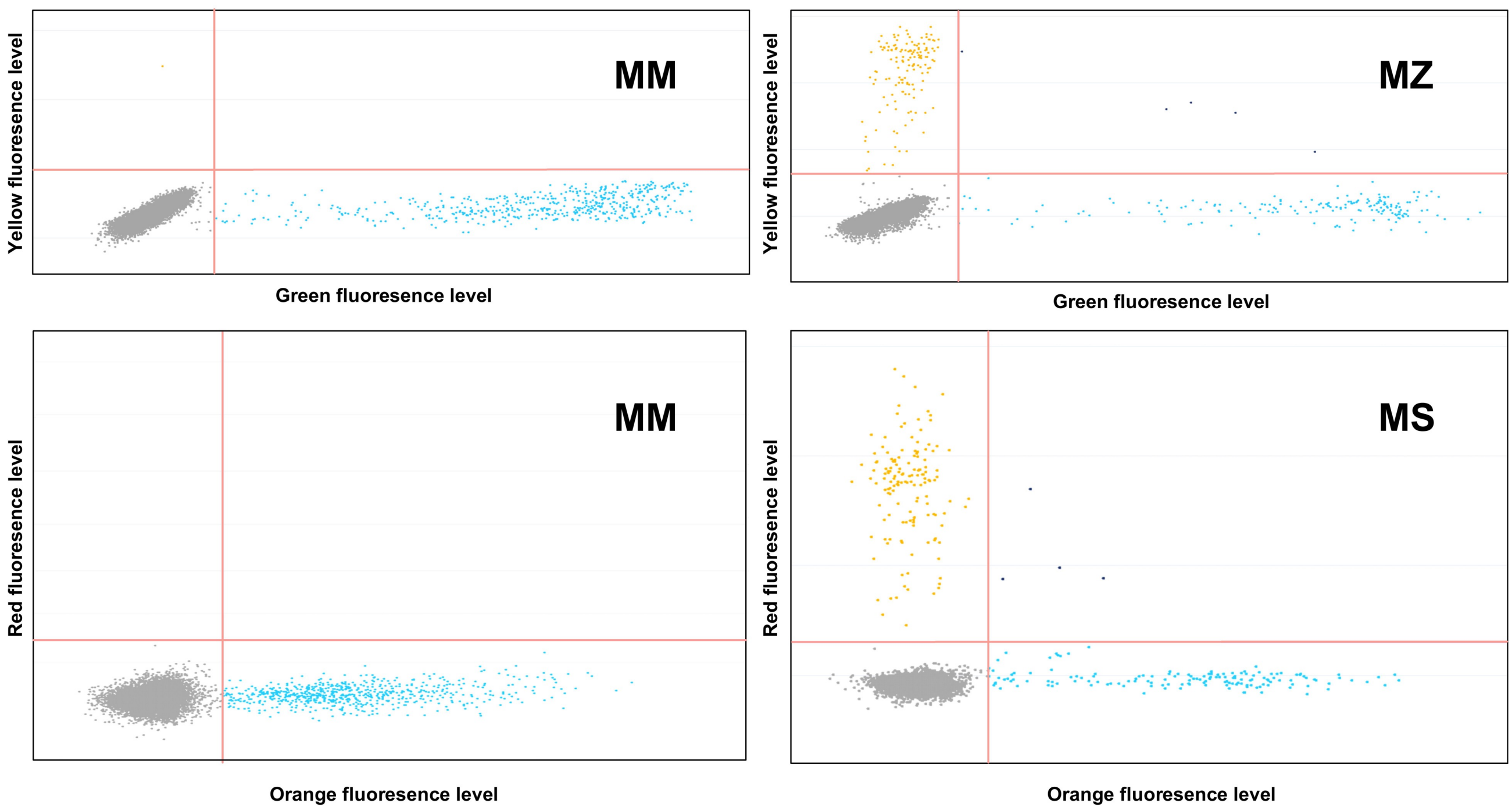

Supplemental Figure 2

Table S1. List of primers, probes, and fluorophores.

Primer reference	Sequence	PCR product length
SA1-EX5-FWD	5’-GCCTGGGATCAGCCTTACAA-3’	126bp
SA1-EX5-REV	5’-ATGGGTATGGCCTCTAAAAACA-3’	
SA1-EX4-FWD	5’-CTGGGTGCTGCTGATGAAATA-3’	121bp
SA1-EX4-REV	5’-TTCCAGGAACTTGGTGATGATA-3’	
**Probe reference**	**Sequence**	**Fluorophore & quencher**
EX5-WT	5’-ACCATCGAC**G**AGAAAGGGAC-3’	FAM + Eclipse Dark Quencher
EX5-Zmut	5’-ACCATCGAC**A**AGAAAGGGAC-3’	HEX + Eclipse Dark Quencher
EX4-WT	5’-CAGCACCTGG**A**AAATGAACTC-3’	TAMRA + Dark Quencher 2
EX4-Smut	5’-CAGCACCTGG**T**AAATGAACTC-3’	ROX + Dark Quencher 2

bp: base pair; Letters in bold indicate the position of the probe nucleotide recognition site of the variant (wild-type in blue, mutated in red)

Table S2: Histopathological features of cohort AATD patients

	AATD patients (n=19)	
**Factors**	**Category**	**n (%)**
**WHO Tumor grade**	1	8 (42)
	2	8 (42)
	3	2 (11)
**Histological subtypes**	NOS	12 (63)
	Steato-hepatitic	2 (11)
	Clear cells	2 (11)
	Macrotrabecular-massive	1 (5)
	Scirrhous	1 (5)
	Chromophobe	0 (0)
	Neutrophil-rich	0 (0)
	Lymphocyte-rich	0 (0)
**VETC**	<55% of the tumor surface	4 (21)
	≥55% of the tumor surface	1 (5)
**Vascular invasion**	Yes	8 (42)
	No	11 (58)
**Tumor fibrosis**	No or weak	13 (68)
	Moderate	2 (11)
	Marked	3 (16)
**Tumor inflammation**	No or weak	14 (74)
	Moderate	3 (16)
	Marked	1 (5)
**Intracellular globules on PAS-diastase staining**	In the tumor	0 (0)
	In the non-tumoral liver	7 (37)
**Non-tumoral liver fibrosis**	F0	4 (21)
	F1	7 (37)
	F2	2 (11)
	F3	3 (16)
	F4	0 (0)

VETC, vessels encapsulating tumor clusters
